# Supplementary material for: Methyl Jasmonate Treatment of Broccoli Enhanced Glucosinolate Concentration, Which Was Retained after Boiling, Steaming, or Microwaving
Source: Foods. 2020 Jun 8;9(6):758. doi: 10.3390/foods9060758 (PMC7353551; doi:10.3390/foods9060758)
Supplement: Supplementary file 1 [file foods-09-00758-s001.pdf]

Table S1. Total glucosinolate profile (μmole·mL) in the cooking water collected from cooked broccoli with or without 250 μM methyl jasmonate application (average ± SD, n=3).

			Total glucosinolates in cooking water (μmole·mL)
Boiling	2 min	Control	0.10±0.00
		MeJA	0.22±0.30 ^{ns}
	5 min	Control	0.13±0.01
		MeJA	0.05±0.00*
Steaming	2 min	Control	0.01±0.00
		MeJA	0.01±0.00 ^{ns}
	5 min	Control	0.01±0.01
		MeJA	0.02±0.00*
Microwaving	2 min	Control	0.03±0.01
		MeJA	0.01±0.00 ^{ns}
	5 min	Control	0.03±0.01
		MeJA	0.08±0.02*

Asterisk (*) indicates significant difference with or without MeJA within the same cooking treatment by Student's T-test (P≤0.05, N=3). ^{ns}, not significant; *, P≤0.05; **, P≤0.01; ***, P≤0.001.

Table S2. Results of two-way ANOVA (MeJA treatment × cooking method) for glucosinolate profiles of 2-minute cooking (A) and of 5-minute cooking (B).

(A)

Total Aliphatic GLS					
Source	Number of parameters	DF	Sum of Squares	F Ratio	Prob > F
MeJA treatment	1	1	2.55258	2.0434	0.1721
Cooking	3	3	79.07297	21.1	<.0001
Cooking*MeJA	3	3	9.954825	2.6564	0.0837
**Total indolyl GLS**					
Source	Number of parameters	DF	Sum of Squares	F Ratio	Prob > F
MeJA treatment	1	1	3278.25	464.2703	<.0001
Cooking	3	3	639.8043	30.2033	<.0001
Cooking*MeJA	3	3	260.3897	12.2923	0.0002
**Total GLS**					
Source	Number of parameters	DF	Sum of Squares	F Ratio	Prob > F
MeJA treatment	1	1	2790.209	319.1227	<.0001
Cooking	3	3	984.7455	37.5425	<.0001
Cooking*MeJA	3	3	353.2774	13.4684	0.0001

(B)

Total Aliphatic GLS					
Source	Nparm	DF	Sum of Squares	F Ratio	Prob > F
MeJA treatment	1	1	2.552580	28.3724	0.1721
Cooking	3	3	79.072965	19.1566	<.0001
Cooking*MeJA	3	3	9.954825	5.718	0.0834
**Total indolyl GLS**					
Source	Nparm	DF	Sum of Squares	F Ratio	Prob > F
MeJA treatment	1	1	3278.25	464.2703	<.0001
Cooking	3	3	639.8043	30.2033	<.0001
Cooking*MeJA	3	3	260.3897	12.2923	0.0002
**Total GLS**					
Source	Nparm	DF	Sum of Squares	F Ratio	Prob > F
MeJA treatment	1	1	3262.142	280.3611	<.0001
Cooking	3	3	1153.127	33.0347	<.0001
Cooking*MeJA	3	3	320.7688	9.1894	0.0009

Table S3. Glucosinolate profile (μmole • g^{-1} dry weight) of aliphatic glucosinolates (A) and indole and benzenic glucosinolates (B) in raw cooked broccoli with or without 250 μM methyl jasmonate application (average ± SD, n=3). same cooking treatment.

(A)

			Glucoraphanin	Gluconapin	Progoitrin	Glucoerucin	Glucoiberin	Sinigrin	Total Aliphatic GSLs
Raw		Control	3.30±0.29	0.09±0.02	0.47±0.05	0.06±0.01	0.56±0.05	0.15±0.01	4.63±0.39
		MeJA	2.33±0.25*	0.06±0.01*	0.56±0.06*	0.08±0.01*	0.43±0.05*	0.14±0.01 ns	3.59±0.38*
Boiling	2 min	Control	1.75±0.23	0.31±0.03	0.29±0.07	0.03±0.00	0.45±0.10	0.08±0.02	2.92±0.41
		MeJA	1.76±0.18 ns	0.34±0.02 ns	0.40±0.04 ns	0.03±0.00*	0.48±0.07 ns	0.11±0.01 ns	3.13±0.08 ns
	5 min	Control	1.41±0.09	0.20±0.06	0.20±0.01	0.03±0.00	0.33±0.03	0.06±0.01	2.23±0.08
		MeJA	1.14±0.19 ns	0.17±0.23 ns	0.28±0.05 ns	0.01±0.01*	0.23±0.05 ns	0.07±0.01 ns	1.90±0.55 ns
Steaming	2 min	Control	2.64±0.35	0.09±0.01	0.44±0.03	0.05±0.01	0.46±0.07	0.15±0.01	3.83±0.45
		MeJA	2.45±0.11 ns	0.07±0.01 ns	0.61±0.02*	0.08±0.01*	0.45±0.02 ns	0.15±0.01 ns	3.81±0.14 ns
	5 min	Control	3.00±0.02	0.08±0.03	0.45±0.04	0.06±0.01	0.54±0.00	0.15±0.02	4.27±0.12
		MeJA	2.47±0.36 ns	0.07±0.01 ns	0.61±0.07*	0.08±0.00*	0.48±0.09 ns	0.15±0.03 ns	3.86±0.57 ns
Microwaving	2 min	Control	2.63±0.52	0.09±0.02	0.36±0.05	0.05±0.01	0.45±0.10	0.13±0.03	3.72±0.72
		MeJA	2.17±0.21 ns	0.06±0.00 ns	0.53±0.06*	0.06±0.01 ns	0.40±0.04 ns	0.13±0.02 ns	3.34±0.33 ns
	5 min	Control	2.75±0.15	0.08±0.00	0.40±0.05	0.06±0.01	0.51±0.04	0.14±0.01	3.94±0.24
		MeJA	2.54±0.13 ns	0.08±0.02 ns	0.68±0.08*	0.08±0.01*	0.49±0.05 ns	0.17±0.02 ns	4.03±0.29 ns

Asterisk (*) indicates significant difference with or without MeJA within the same cooking treatment by Student's T-test (*P*≤0.05, *N*=3) with the significant interaction between MeJA treatment and cooking treatment detected (Supplementary Table S2). ns, not significant; *, *P*≤0.05

(B)

			Glucobrassicin	Neoglucobrassicin	4-methoxy-glucobrassicin	Total indole GSLs	Gluconasturtiin	Total GSLs
Raw		Control	1.52±0.09	2.50±0.24	0.25±0.02	4.26±0.32	0.05±0.01	8.93±0.63
		MeJA	2.31±0.31*	14.68±1.97*	0.30±0.03*	14.21±6.15*	0.05±0.01 ns	20.92±2.62*
Boiling	2 min	Control	0.90±0.02	1.10±0.04	0.15±0.00	2.19±0.07	0.04±0.01	5.16±0.35
		MeJA	1.79±0.06*	7.07±0.16*	0.20±0.01*	9.10±0.23*	0.07±0.04 ns	12.30±0.18*
	5 min	Control	0.66±0.04	0.81±0.11	0.11±0.01	1.61±0.16	0.04±0.01	3.88±0.17
		MeJA	1.48±0.06*	6.27±1.45*	0.15±0.01*	7.93±1.45*	0.03±0.01 ns	9.86±0.93*
Steaming	2 min	Control	1.59±0.07	2.34±0.22	0.25±0.00	4.19±0.30	0.05±0.00	8.06±0.21
		MeJA	2.34±0.08*	14.83±1.35*	0.32±0.01*	17.50±1.44*	0.16±0.05*	21.47±1.51*
	5 min	Control	1.44±0.06	2.02±0.21	0.23±0.01	3.09±1.12	0.05±0.01	8.03±0.27
		MeJA	2.49±0.14*	15.53±1.40*	0.30±0.01*	18.33±1.46*	0.09±0.01*	22.29±1.73*
Microwaving	2 min	Control	1.37±0.24	2.02±0.35	0.24±0.04	3.62±0.61	0.03±0.00	7.38±1.29
		MeJA	2.33±0.17*	14.10±1.73*	0.30±0.03 ns	16.73±1.83*	0.04±0.01 ns	20.12±2.14*
	5 min	Control	1.44±0.08	2.16±0.29	0.23±0.02	3.82±0.37	0.04±0.01	7.81±0.51
		MeJA	2.33±0.08*	15.08±1.67*	0.31±0.03*	17.73±1.73*	0.08±0.03 ns	21.85±1.83*

Asterisk (*) indicates significant difference with or without MeJA within the same cooking treatment by Student's T-test (P≤0.05, N=3) with the significant interaction between MeJA treatment and cooking treatment detected (Supplementary Table S2). ns, not significant; *, P≤0.05

Table S4. Two-way analysis of variance (ANOVA) of the electrical conductivity in cooking water of broccoli with or without 250 μM MeJA treatment.

**2- min cooking**					
**Source**	Nparm	DF	Sum of Squares	F Ratio	Prob > F
**MeJA treatment**	1	1	254.4	0.5095	0.489
**Cooking**	2	2	2709778.6	2713.816	<.0001
**Cooking*MeJA**	2	2	768.7	0.7698	0.4847
**5-min cooking**					
**Source**	Nparm	DF	Sum of Squares	F Ratio	Prob > F
**MeJA treatment**	1	1	12675.9	10.7719	0.0066
**Cooking**	2	2	4322252	1836.512	<.0001
**Cooking*MeJA**	2	2	6115.6	2.5985	0.1154

Table S5. The peak intensity (×10³) of glucosinolate hydrolysis products in (A) raw and 2-minute cooked and (B) raw and 5-minute cooked "Green Magic" broccoli with or without 250 μM methyl jasmonate application (average ± SD, n=3). Compound identification were based on a previous publication (Kim et al., 2017) or by comparison with data in the National Institute of Standards and Technology (NIST) library.

(A)

	**Raw**		**2-minute Boiling**		**2-minute Steaming**		**2-minute Microwaving**	
	**Control**	**MeJA**	**Control**	**MeJA**	**Control**	**MeJA**	**Control**	**MeJA**
Sulforaphane	0.10±0.02	0.39±0.19 ns	0.08±0.07	0.27±0.13 ns	0.12±0.12	0.20±0.17 ns	0.10±0.09	0.11±0.07 ns
Sulforaphane nitrile	4.37±1.03	4.58±1.48 ns	5.47±0.39	3.51±0.95 ns	26.02±30.61	3.84±0.73*	8.60±6.99	2.36±1.78 ns
Erucin nitrile	1.25±0.75	0.67±0.81 ns	0.28±0.34	1.83±2.66 ns	0.38±0.33	0.31±0.19 ns	0.17±0.18	3.13±1.17**
1-cyano-3,4 epithiobutene	0.78±0.43	0.37±0.09 ns	0.39±0.26	0.60±0.20 ns	0.73±0.77	0.29±0.05 ns	0.91±0.70	0.33±0.14 ns
1-Cyano-2-hydroxy-3,4-epithiobutane	0.82±0.42	2.16±0.55**	1.46±1.24	0.67±0.25 ns	0.98±0.83	0.72±0.12 ns	0.59±0.10	0.55±0.31 ns
NMI3CA	35.00±11.30	116.13±38.96***	5.89±0.76	13.70±3.36 ns	22.75±5.79	35.26±8.82 ns	2.86±0.83	7.25±0.38 ns
NMI3CAN	0.34±0.33	0.65±0.17***	0.29±0.22	0.69±0.30 ns	0.49±0.14	0.44±0.12 ns	0.49±0.10	0.15±0.18**
NMI3C	450.48±39.56	3991.30±539.36***	65.76±8.96	53.09±47.95 ns	106.48±95.03	4.13±5.58 ns	33.30±31.54	523.48±67.09**
Indole-3-acetonitrile	0.21±0.14	0.26±0.36 ns	0.25±0.24	0.48±0.05 ns	0.78±0.35	1.89±1.11**	0.31±0.03	0.44±0.16 ns
Indole-3-carbinol	0.23±0.12	0.93±0.13***	0.33±0.15	0.67±0.20*	0.34±0.22	0.45±0.19 ns	0.21±0.17	0.22±0.11 ns
Phenylethyl ITC	0.44±0.23	1.23±0.26	3.80±0.18	1.80±0.40 ns	2.13±2.74	3.43±1.31 ns	1.97±1.22	1.83±1.51 ns
Total Peak Intensity	494.85±50.28	4120.84±579.74***	83.72±8.20	77.72±47.80 ns	163.12±83.80	51.59±12.22 ns	49.66±36.61	540.69±70.06***

(B)

	Raw		5-minute Boiling		5-minute Steaming		5-minute Microwaving	
	**Control**	**MeJA**	**Control**	**MeJA**	**Control**	**MeJA**	**Control**	**MeJA**
Sulforaphane	0.10±0.02	0.39±0.19	0.05±0.07	0.12±0.10**	0.14±0.08	0.23±0.15	0.17±0.06	0.20±0.05
Sulforaphane nitrile	4.37±1.03	4.58±1.48 ^{ns}	3.64±0.39	9.31±3.78**	4.40±2.10	3.45±0.98 ^{ns}	8.00±6.96	4.76±1.48 ^{ns}
Erucin nitrile	1.25±0.75	0.67±0.81 ^{ns}	0.83±0.34	0.86±0.97 ^{ns}	0.58±0.25	0.50±0.25 ^{ns}	0.45±0.27	0.31±0.14 ^{ns}
1-cyano-3,4 epithiobutene	0.78±0.43	0.37±0.09 ^{ns}	0.34±0.26	0.63±0.20 ^{ns}	0.34±0.18	0.08±0.06 ^{ns}	0.31±0.23	0.23±0.24 ^{ns}
1-Cyano-2-hydroxy-3,4-epithiobutane	0.82±0.42	2.16±0.55 ***	1.15±0.26	0.65±0.20 ^{ns}	0.38±0.18	0.70±0.06 ^{ns}	0.34±0.23	0.75±0.24 ^{ns}
NMI3CA†	35.00± 11.30	116.13± 38.96*	3.82±0.76	15.69±4.73 ^{ns}	56.13±25.34	78.29±41.51*	28.35±10.68	101.45± 46.64**
NMI3ACN ‡	0.34±0.33	0.65±0.17 ***	0.54±0.22	0.25±0.42 ^{ns}	0.60±0.58	0.77±1.23 ^{ns}	0.55±0.45	0.46±0.42**
NMI3C §	450.48± 39.56	3991.30± 539.36***	599.27±8.96	812.66± 371.75 ^{ns}	135.23± 231.83	182.61± 55.04**	180.02±114.76	2361.27± 1006.49 **
Indole-3-acetonitrile	0.21±0.14	0.26±0.36 ^{ns}	0.37±0.24	0.23±0.30 ^{ns}	0.61±0.45	3.66±5.01 ^{ns}	0.23±0.23	0.51±0.20 ^{ns}
Indole-3-carbinol	0.23±0.12	0.93±0.13 ***	0.36±0.15	0.33±0.09 ^{ns}	0.39±0.23	0.25±0.11 ^{ns}	0.31±0.13	0.72±0.41*
Phenylethyl ITC	0.44±0.23	1.23±0.26 ^{ns}	3.19±0.18	1.24±0.48 ^{ns}	4.08±2.36	3.37±1.75*	3.04±2.85	2.06±0.92 ^{ns}
Total Peak Intensity	494.43± 50.06	4119.09± 579.83***	614.72± 8.20	842.64± 371.75 ^{ns}	204.44± 234.45	274.67± 78.81 ^{ns}	222.11± 100.33	2473.47± 1054.43*

†NMI3CA== *N*-methoxyindole-3- carboxyaldehyde; ‡NMI3CN= *N*-methoxyindole-3-acetonitrile; §NMI3C= *N*-methoxyindole-3-carbinol. Asterisk (*) indicates significant difference with or without MeJA within the same cooking treatment by Student's T-test (P≤0.05, N=3). ^{ns}, not significant; *, P≤0.05; **, P≤0.01; ***, P≤0.001.

Table S6. Primary metabolites that high in variable influence on projection (VIP) by partial least square – discrimination analysis (PLS-DA) from raw and cooked 'Green Magic' broccoli with or without 250 μM MeJA treatment by GC-MS analysis. All listed metabolites were significant changed by ANOVA at $P \leq 0.05$.

Tentative metabolites	Retention time (min)	Characteristic ion (*m/z*)	TMS [‡]	VIP	Identification
Oxoproline	9.57	73,147,**156** [†]	$(TMS)_2$	5.94	NIST
Glutamic acid	10.36	73,128,**246**	$(TMS)_3$	1.89	STD/NIST[w]
Valine	6.75	73,**144**,218	$(TMS)_2$	1.69	STD/NIST
Proline	7.58	73,**142**,216	$(TMS)_2$	2.85	STD/NIST
Serine	7.17	73,**116**,132	$(TMS)_2$	1.97	STD/NIST
Isoleucine	7.52	73,**158**,218	$(TMS)_2$	2.09	STD/NIST
Alanine	5.60	73, **116**, 147	$(TMS)_2$	2.70	(Chiu, Juvik, & Ku, 2018)
Fructose	12.3	73, **103**, 147, 217, 307	Meox, $(TMS)_5$	1.93	(Chiu, Juvik, & Ku, 2018)
Glucose[y]	12.42	**73**, 147, 160, 205, 319	Meox, $(TMS)_5$	8.11	(Chiu, Juvik, & Ku, 2018)
Sucrose	16.80	73, 147, 217, 271, **361**	$(TMS)_8$	1.63	(Chiu, Juvik, & Ku, 2018)
Myo-Inositol	13.73	73**, 147**, 217, 305, 318	$(TMS)_6$	1.98	STD/NIST
Quinic acid	12.15	147,255,**345**	$(TMS)_5$	1.66	NIST

[†] Highest peak is label as bold

[‡] Meox, methyloxime; TMS, trimethylsilyl.

[§] Metabolites were identified using commercial standard compounds (STD) in comparison with the mass spectra in The National Institute of Standards and Technology (NIST) and retention time.
